# DTD: Density Triangle Descriptor for 3D LiDAR Loop Closure Detection

**DOI:** 10.3390/s26010201

**Published:** 2025-12-27

**Authors:** Kaiwei Tang, Qing Wang, Chao Yan, Yang Sun, Shengyi Liu

**Affiliations:** 1 School of Instrument Science and Engineering, Southeast University, Nanjing 210096, China; 220233592@seu.edu.cn (K.T.); yang_sun@seu.edu.cn (Y.S.); liusy_seu@seu.edu.cn (S.L.); 2 School of Electrical Engineering and Automation, Suzhou University of Technology, Suzhou 215500, China

**Keywords:** loop closure detection, LiDAR SLAM, density image, point cloud descriptor

## Abstract

Loop closure detection is essential for improving the long-term consistency and robustness of simultaneous localization and mapping (SLAM) systems. Existing LiDAR-based loop closure approaches often rely on limited or partial geometric features, restricting their performance in complex environments. To address these limitations, this paper introduces a Density Triangle Descriptor (DTD). The proposed method first extracts keypoints from density images generated from LiDAR point clouds, and then constructs a triangle-based global descriptor that is invariant to rotation and translation, enabling robust structural representation. Furthermore, to enhance local discriminative ability, the neighborhood around each keypoint is modeled as a Gaussian distribution, and a local descriptor is derived from the entropy of its probability distribution. During loop closure detection, candidate matches are first retrieved via hash indexing of triangle edge lengths, followed by entropy-based local verification, and are finally refined by singular value decomposition for accurate pose estimation. Extensive experiments on multiple public datasets demonstrate that compared to STD, the proposed DTD improves the average F1 max score and EP by 18.30% and 20.08%, respectively, while achieving a 50.57% improvement in computational efficiency. Moreover, DTD generalizes well to solid-state LiDAR with non-repetitive scanning patterns, validating its robustness and applicability in complex environments.

## 1. Introduction

Loop closure detection (LCD) is a fundamental component of simultaneous localization and mapping (SLAM) systems, aiming to determine whether the robot’s current location corresponds to a previously visited place. By establishing data associations between the current frame and historical loop candidates, LCD effectively corrects accumulated localization drift, thereby significantly improving the accuracy of pose estimation and the consistency of the global map. This mechanism has been widely integrated into SLAM systems such as LIO-SAM [1], ORB-SLAM [2], and LV-SLAM [3]. Based on the type of sensor employed, LCD methods can be broadly categorized into vision-based and LiDAR-based approaches. In recent years, vision-based place recognition methods have been extensively studied and applied [4,5,6]. However, these methods often suffer from significant limitations in scenarios with severe illumination changes or rapid motion. In contrast, LiDAR-based methods directly capture the 3D structural information of the environment through laser pulses, making them inherently more robust to changes in lighting and viewpoint. Most existing LiDAR-based loop closure detection methods adopt descriptor-based frameworks. Representative approaches such as M2DP [7] and Scan Context [8] rely on projection-based representations to encode global structural information in point clouds. More recently, several methods have further enhanced descriptor discriminability by incorporating additional cues, such as visual features during keypoint extraction [9] or reflectivity information in RE-TRIP [10], thereby improving robustness in complex environments. Inspired by the above research progress, this paper focuses on the loop closure detection problem based on LiDAR, aiming to design a descriptor that is both efficient and robust to adapt to diverse scenarios.

Compared to camera images, LiDAR point clouds are more unstructured and complex, which poses challenges for place recognition. (1) First, point clouds are sensitive to the sensor’s position and orientation. To handle viewpoint changes, LiDAR-based methods should achieve rotation and translation invariance, and several geometric descriptors such as triangles [11,12], Link3D [13], and rings [14] have been proposed. (2) Second, the descriptor must effectively capture the structural features of the environment in a compact form. (3) Finally, the descriptor should be robust enough to support relative pose estimation between the current frame and loop candidates, helping reduce drift in mapping and localization.

To address these challenges, we propose Density Triangle Descriptor (DTD), a spatial descriptor based on density images. DTD extracts keypoints from density BEV (Bird’s Eye View) images, restores them to 3D coordinates, and connects them using triangles. In addition, to enhance the representation of local structures around each keypoint, the probabilistic distribution of neighboring points is modeled as a local descriptor. Specifically, our contributions are as follows:

1. We propose a novel Density Triangle Descriptor (DTD) based on density image features, which enables fast keypoint extraction from point cloud density maps and can be robustly applied to loop closure detection across various scenarios.
2. We introduce an entropy-based local descriptor, in which the neighborhood of each keypoint is modeled as a Gaussian distribution, and entropy is employed to quantify local uncertainty, thereby enriching the expressiveness of DTD.
3. We develop a two-stage loop closure detection method for DTD, consisting of candidate search and geometric verification, where an entropy-based similarity function is further incorporated to improve the real-time performance and robustness of the method.
4. Extensive experiments across multiple scenarios demonstrate the effectiveness of our method, with results compared to state-of-the-art approaches.

## 2. Related Work

Current loop closure detection approaches can be broadly categorized into Vision-based and LiDAR-based methods. The method proposed in this work integrates both visual-inspired cues and LiDAR texture information. In the following, we provide a review of the related literature.

### 2.1. Vision-Based Loop Closure Detection

Vision-based LCD has been widely applied in various SLAM systems, with Bag-of-Words (BoW) models being among the most commonly used due to their efficiency and generalization capability. These approaches typically rely on retrieving 2D image features, where classical descriptors such as SIFT [15], SURF [16], and ORB [17] offer invariance to rotation and translation. For instance, DBoW2 [4] builds binary descriptors based on BRIEF [18], significantly improving the efficiency of image retrieval. FAB-MAP [19] further extends the BoW model by introducing a probabilistic generative framework.

However, these methods mainly exploit the appearance features of 2D images and are prone to false positives in the presence of local structural repetition. To mitigate this, our method not only utilizes the keypoints extracted from LiDAR-based density maps but also preserves their 3D spatial information, thereby reducing the impact of locally similar structures.

### 2.2. LiDAR-Based Loop Closure Detection

In recent years, LiDAR-based LCD has received increasing attention, and existing methods can be broadly classified into handcrafted feature-based and learning-based approaches.

Handcrafted feature-based methods often utilize projection strategies to generate low-dimensional descriptors. A representative example is M2DP [7], which projects point clouds onto multiple 2D planes to generate density signatures, and then applies singular value decomposition (SVD) to derive global descriptors. Another widely adopted method is Scan Context (SC) [8], which projects point clouds into BEV space under a polar coordinate system and encodes each bin using the maximum height, thereby achieving rotation invariance. However, SC lacks translation invariance. SC++ [20] improves upon SC by enhancing robustness to lateral shifts and rotational movements while also increasing search efficiency. Several SC variants have also been proposed to encode point clouds using alternative feature cues [21,22,23]. NDD [24] employs a probabilistic 2D representation to better capture the density distribution of point clouds. BoW3D [13] constructs a bag-of-words representation using Link3D features, enabling efficient place recognition and real-time 6-DoF loop pose correction. More recently, STD [11] has been introduced by Yuan et al., which extracts keypoints and constructs triangle-based descriptors with both rotation and translation invariance. However, since STD relies on planar structures for keypoint extraction, it suffers from reduced efficiency and limited discriminability in low-texture environments.

Learning-based methods have also emerged with the advancement of deep learning. PointNetVLAD [25] combines PointNet [26] and NetVLAD [27] to extract global point cloud features with high matching accuracy. OverLapNet [28] proposes a lightweight architecture to extract multi-scale features and estimates the relative yaw angle and overlap between scans. SSC [29] leverages semantic information to encode 3D scenes and incorporates a two-stage global ICP strategy to improve registration performance. BEVPlace++ [30] further introduces a rotation equivariant and invariant network (REIN), achieving robust place recognition under significant rotational variations. Although learning-based methods show promising performance, they typically require large-scale annotated datasets and substantial computational resources, making them less suitable for real-time deployment on general platforms. Therefore, this work focuses on handcrafted descriptors that are training-free and computationally efficient.

To balance feature expressiveness and environmental adaptability, we propose a loop closure detection framework that integrates both visual and LiDAR information. Our method generates 3D keypoints based on density map projections and constructs global descriptor through triangle topology, enhancing structural representation and matching stability. Additionally, entropy-based local descriptor is designed to characterize the local neighborhood of each keypoint using the probabilistic distribution of point density, thereby improving local discriminability.

## 3. Methods

**Figure 1:**
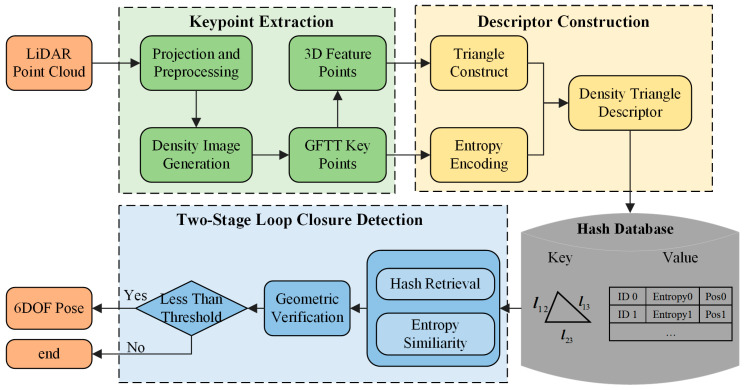
The pipeline of the DTD framework. The approach extracts 2D GFTT keypoints from the density BEV image, constructs triangular structures in 3D space to encode global geometry, and employs entropy to describe local distributions. During loop closure detection, candidate frames are retrieved via triangle-edge hash search and entropy similarity, followed by geometric verification to confirm true loops and estimate relative poses.

In this section, we present the overall framework of the proposed Density Triangle Descriptor (DTD), as illustrated in Figure 1. The pipeline begins with the keypoint extraction module, where the input point cloud is first preprocessed and keypoints are detected from the density-based BEV image. This is followed by the descriptor construction module, in which a descriptor is formed from three selected keypoints. The descriptor integrates both the geometric side lengths of the triangle and the probabilistic distribution characteristics of the keypoint neighborhoods. Finally, a two-stage loop closure detection algorithm is introduced, which integrates global geometric representation from triangle edge lengths with local entropy-based descriptors, ultimately yielding a 6-DoF pose estimation.

### 3.1. Keypoint Extraction

Bird’s Eye View (BEV) projection is widely used in place recognition because it preserves local 2D geometric structure while reducing computational cost through dimensionality reduction. Traditional BEV images built from maximum height [8] or intensity [31] are often sensitive to pose variations or external factors such as reflectivity and sensor noise. In contrast, density-based BEV representations [32] rely on point distributions within grid cells rather than raw point values, offering better robustness to viewpoint changes. Motivated by this advantage, we adopt a density image as the BEV representation in this work.

**Figure 2:**
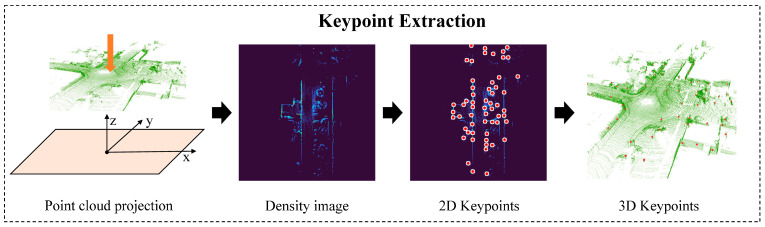
Workflow of keypoint extraction. The input point cloud is first preprocessed and projected into a density image, from which GFTT keypoints are detected. These keypoints are then back-projected to obtain their corresponding 3D positions.

Specifically, the BEV plane is uniformly partitioned into square grid cells of equal area, upon which the density distribution is computed. The raw LiDAR point cloud is defined as follows:

(1) $$P=\{p_{1},p_{2},\ldots ,p_{n}\;|\;p_{i}\in {\mathbb{R}}^{3}\}$$

where  $p_{i}$  denotes a point in the 3D space. In typical road environments, the x, y, and z axes point toward the right, forward, and upward directions of the LiDAR sensor, respectively, with the x−y plane corresponding to the ground. Centered on the LiDAR, we define a square projection region covering  $\left[-L\;m,\;L\;m\right]$  in the x–y plane, as illustrated in Figure 2 (point cloud projection). A keyframe is formed by accumulating a fixed number of LiDAR scans; the aggregated point cloud is then discretized into an  M×M  grid. For each grid cell, the number of points falling within it is counted as  C(u,v)  based on the x and y coordinates of the points. These counts are subsequently normalized to obtain the density image  I(u,v), defined as follows:

(2) $$I(u,\nu )=\left\{\begin{array}{l} \frac{C(u,\nu )-C_{\min}}{C_{\max}-C_{\min}}|\alpha -\beta |+\min(\alpha ,\beta )\;\mathrm{if}\;\frac{I(u,\nu )}{I_{\max}}\geq \sigma_{k}\\ \;\;\;0\;\;\;\;\;\;\;\;\;\;\;\;\;\;\mathrm{if}\;\frac{I(u,\nu )}{I_{\max}}<\sigma_{k} \end{array}\right.$$

(3) $$C_{\max}=\max C(u,v);C_{\min}=\min C(u,v);I_{\max}=\max I(u,v).$$

where  α  and  β  control the range of normalized values. Furthermore, a proportional threshold  $\sigma_{k}$ is applied to the density image in this study, where all pixels with density values below this threshold are set to zero. This design effectively suppresses the influence of ground points and noise in the density image generation process, thereby ensuring greater consistency and reliability in the subsequent keypoint extraction.

After generating the density image, we perform feature extraction to identify salient regions. Density image typically contains rich local textures and pronounced structural variations. Since the sensor may undergo significant changes in orientation during vehicle motion, it is essential for the extracted features to exhibit rotational invariance. To this end, we adopt the Good Features to Track (GFTT) algorithm [33] to detect corner features within the density image.

The GFTT algorithm effectively captures salient characteristics in the point cloud density distribution, especially in areas with strong local structural variations. The detected 2D feature points are then projected back into 3D space by computing the mean coordinates of the raw 3D points within their corresponding grid cells (as illustrated in Figure 2). This process yields the keypoints for each keyframe, effectively combining the local structural information of the density map with the spatial properties of the point cloud, and providing a robust foundation for subsequent descriptor generation.

### 3.2. Density Triangle Descriptor Construction

We construct triangle descriptors using the keypoints generated in Section 3.1. Owing to the inherent translation and rotation invariance of triangles, a set of triangles formed by keypoints can effectively represent the global geometric structure of each keyframe. To ensure the uniqueness and geometric validity of the triangles, we employ a kd-tree to search for two neighboring points for each keypoint so that each triplet forms only one triangle. Additionally, the side lengths of triangles are constrained within a predefined threshold range to avoid overly sparse or dense connections.

When describing local point cloud features, many existing methods rely on simple attributes such as height, intensity, or density, which often lack robustness in complex outdoor environments. Since each vertex of a triangle corresponds to a grid cell, we model the point distribution within each cell as a Gaussian distribution  x ~ N(μ,Σ), where  μ is the mean and  Σ is the full covariance matrix. We compute its uncertainty using information entropy, the entropy vector for the three vertices is defined as  $H=(H_{1},H_{2},H_{3})$, where

(4) $$\begin{array}{c} H(x)=-\sum_{i=1}^{n}P(x_{i})\log P(x_{i})\\ \;=\frac{1}{2}\log({\left(2\pi e\right)}^{d}\;|\Sigma |) \end{array}$$

where  d represents the dimension of the data, which is set to 3 for a 3D point cloud. This probabilistic modeling based on Gaussian statistics effectively reduces the influence of outliers and improves local feature stability.

The proposed Density Triangle Descriptor combines density and entropy information to jointly capture both the spatial density and uncertainty of local regions. Each descriptor  D consists of the vertex coordinates  $\left(p_{1},p_{2},p_{3}\right)$, side lengths  $\left(l_{12},l_{23},l_{13}\right)$, the corresponding entropy values  $\left(h_{1},h_{2},h_{3}\right)$, and the frame index  k:

(5) $$D=(p_{1},p_{2},p_{3},l_{12},l_{23},l_{13},h_{1},h_{2},h_{3},k)$$

**Figure 3:**
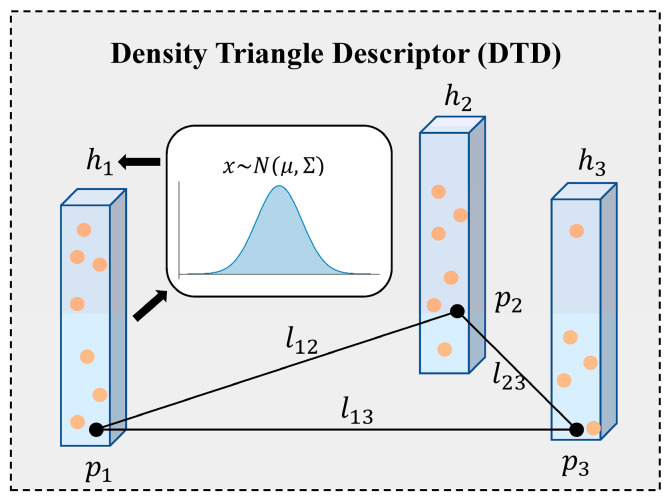
Illustration of the proposed DTD. A triangle is formed by three geometrically valid keypoints to capture global structural features, while entropy of the local neighborhood encodes point-wise local characteristics.

An example of DTD is shown in Figure 3. Finally, each descriptor is indexed into a database using a hash key derived from its side lengths.

### 3.3. Loop Detection

In this section, we propose a two-stage loop closure detection algorithm based on the DTD, as outlined in Algorithm 1. The method consists of the following two stages:
**Algorithm 1:** Loop Closure Detection
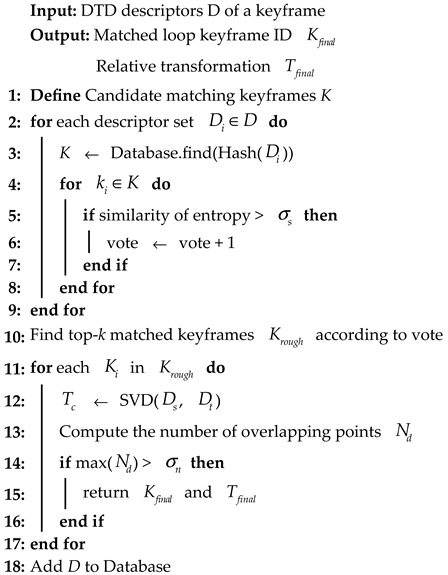


(1) Candidate Search: We first extract global and local descriptors from the current frame using the method described in Section 3.2. For each descriptor, a unique hash key is generated based on the triangle’s edge lengths. These hash keys are then used to query a preconstructed hash table to retrieve candidate descriptors from past frames that exhibit similar triangular structures.

To further refine the candidate set and identify the most relevant loop candidates, we define a local descriptor similarity function, which quantitatively evaluates the similarity between the current local descriptor  $H_{c}$ and a candidate loop local descriptor  $H_{l}$. The similarity is defined as:

(6) $$\mathrm{sim}(H_{c},H_{l})=\frac{H_{c}H_{l}^{T}}{\left\|H_{c}\|\;\|H_{l}\right\|}$$

It can be observed that the similarity function corresponds to the cosine similarity of the entropy vectors. If the similarity between  $H_{c}$ and  $H_{l}$ exceeds a predefined threshold  $\sigma_{s}$, a vote is cast for the frame  $F_{l}$. This voting process is applied to all descriptors in the current frame, and the top-k frames with the highest number of votes are selected as input to the subsequent geometric verification stage. This voting-based strategy effectively narrows down the set of candidate frames, significantly improving both the efficiency and accuracy of the geometric verification process.

(2) Geometric verification: After obtaining the top-k loop candidate frames, we perform geometric verification to eliminate false positives and estimate the 6-DoF relative pose transformation between the current frame and the loop frame. Specifically, for the current keyframe  $F_{c}$ and a candidate loop frame  $F_{l}$, we identify *N* matched descriptor pairs, each corresponding to two point sets  ${\left\{P\right\}}_{c}$ and  ${\left\{P\right\}}_{l}$, respectively. We first compute the centroids  $p_{cc}$ and  $p_{lc}$ of the corresponding point sets, and then construct the cross-covariance matrix:

(7) $$W=\sum_{i=1}^{3*N}\left(p_{c}^{i}-p_{cc}\right){\left(p_{l}^{i}-p_{lc}\right)}^{T}$$

We apply Singular Value Decomposition (SVD) to  W:

(8) $$W=U\Sigma V^{T}$$

If  W is of full rank, the optimal rotation  $R_{c,l}$ and translation  $t_{c,l}$ between the two frames can be recovered as follows:

(9) $$\begin{array}{c} R_{c,l}=VU^{T}\\ \;\;\;t_{c,l}=s_{l}-R_{c,l}s_{c} \end{array}$$

Using this transformation, we project each point  ${\left\{P\right\}}_{l}$ to its estimated location  ${\left\{P\right\}}_{e}$, and compute its Euclidean distance to the corresponding point  ${\left\{P\right\}}_{c}$. A match is considered valid if

(10) $$\|p_{c}^{i}-(R_{c,l}p_{l}^{i}+t_{c,l})\|<\sigma_{d}$$

If the distance is less than  $\sigma_{d}$, the two points are successfully matched. We count the number of valid correspondences, and only if this number  $N_{d}$ exceeds a specified minimum threshold  $\sigma_{n}$, the candidate frame is finally accepted as a valid loop closure.

## 4. Results

### 4.1. Dataset and Experimental Settings

To comprehensively evaluate the loop closure detection performance of the proposed method, experiments were conducted on four publicly available datasets: KITTI [34], NCLT [35], FusionPortable [36], and HeLiPR [37], with detailed information summarized in Table 1.

The KITTI dataset provides ground truth (GT) data corresponding to each point cloud file. In our experiments, each bin file is treated as an individual point cloud frame.

For the NCLT dataset, the timestamps of point cloud data are not strictly aligned with the official GT. Therefore, we employ the FAST-LIO [38] odometry to resample the point clouds at a fixed spatial interval, generating frames at every 1 m of traveled distance.

**Figure 4:**
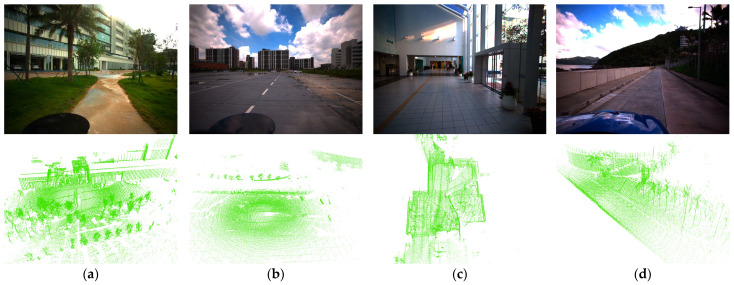
Representative sequences from FusionPortable: (**a**) ugv_transition00, (**b**) ugv_parking01, (**c**) building_day, (**d**) vehicle_campus01. The images above and below are captured by a camera and LiDAR, respectively. The dataset includes indoor–outdoor transitions, open outdoor areas, complex building interiors, and visually similar road segments, which are prone to mismatches.

The FusionPortable dataset contains a variety of complex environments (as shown in Figure 4). Similarly, FAST-LIO is used to process the raw data and generate the corresponding point cloud sequences for evaluation.

The above three datasets are collected using spinning LiDAR sensors. To further investigate the adaptability of our method across different LiDAR modalities, we additionally evaluate our approach on the HeLiPR dataset, which includes data acquired by two solid state LiDAR sensors. The HeLiPR dataset provides GT annotations for each point cloud, and we apply the same 1 m spatial sampling strategy to generate keyframes for experimental validation.

To validate the effectiveness of our method, we compared it against several state-of-the-art approaches. All experiments were executed on a computing platform equipped with an AMD Ryzen 5 5600 @ 3.3 GHz processor and 16 GB RAM, running Ubuntu 20.04 LTS.

### 4.2. Loop Detection Evaluation

In this section, we compare the proposed method with several state-of-the-art (SOTA) approaches, including Scan Context [8] and STD [11]. In our DTD implementation, every 10 consecutive frames are aggregated into a keyframe. A loop closure is considered a true positive when the ground-truth (GT) distance between the target frame and a candidate frame is less than 15 m, with at least 50 keyframes in between to avoid trivial matches.

For parameter settings, when constructing the density image, the projection area range  L is 50, the size of the density image  M is 300, and the density threshold  $\sigma_{k}$ is 0.05. The parameters used for constructing the triangle descriptor are kept consistent with those of STD, while the similarity threshold  $\sigma_{s}$  for the local descriptor is set to 0.95. To evaluate the contribution of the local descriptor, we also compare DTD with its entropy-free variant, DTD (w/o entropy). For Scan Context and STD, we adopt their open-source implementations and followed the recommended parameter settings provided in their respective papers.

**Figure 5:**
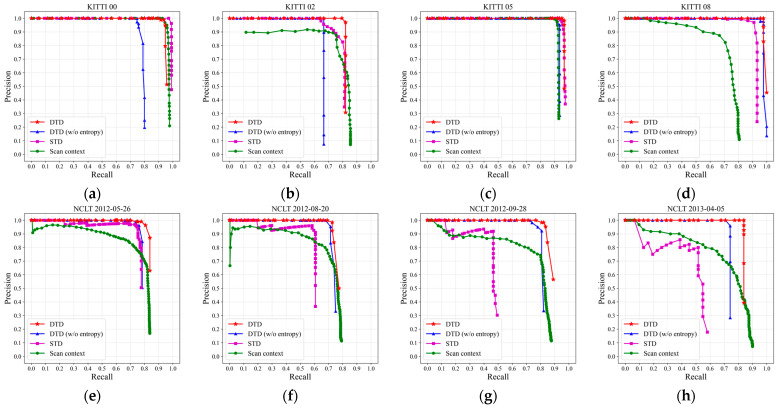

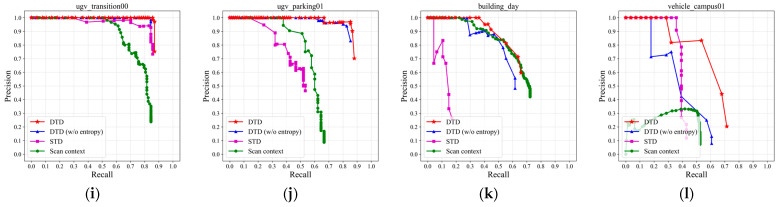
Precision–recall curves for KITTI (**a**–**d**), NCLT (**e**–**h**), and FusionPortable (**i**–**l**) datasets.

By varying the number of coincident points  $N_{d}$, we generate the precision–recall (PR) curves for different sequences (as shown in the Figure 5). Furthermore, we evaluate the F1 max score and the Extended Precision (EP) value of each method (as shown in the Table 1). The F1 score is defined as follows:

(11) $$F_{1}=2\times \frac{P\times R}{P+R}$$

where  P and  R denote precision and recall, respectively. The F1 max score provides a balanced measure between accuracy and completeness. The EP value is defined as follows:

(12) $$EP=\frac{1}{2}\left(P_{R0}+R_{P100}\right)$$

where  $P_{R0}$ represents the precision at minimum recall and  $R_{P100}$ represents the max recall at 100% precision. EP is particularly suitable for evaluating loop closure detection performance.

As shown in Figure 5 and Table 2, DTD consistently achieves superior PR curves, higher F1 max scores, and larger EP values across most sequences. Compared to STD, DTD shows average improvements of 18.30% and 20.08% in F1 max score and EP, respectively; compared to Scan Context, the improvements are 15.51% and 33.40%, respectively.

For the KITTI dataset, DTD (w/o entropy) performs less favorably on KITTI 00 and KITTI 02, as it relies solely on density-based triangles for matching. Incorporating entropy significantly improves the robustness, leading to 12.51% and 11.13% increases in the F1 max score, respectively. In the reverse-loop-only KITTI 08 sequence, Scan Context exhibits degraded performance, whereas DTD remains stable, demonstrating strong rotation invariance.

For the NCLT dataset, which contains diverse indoor–outdoor transitions, both STD and Scan Context show inconsistent results. Even without entropy, DTD (w/o entropy) remains stable, and the integration of entropy further enhances loop detection performance.

**Figure 6:**
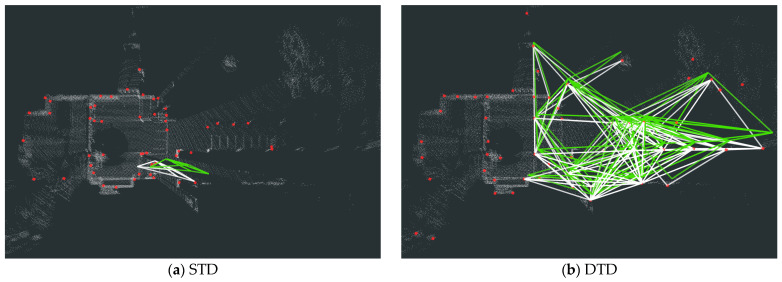
A representative comparison of the matching performance between STD and DTD in the building_day sequence. (**a**) Failed matching with the STD method; (**b**) successful matching with the DTD method. The red dots denote the keypoints, while the white and green lines represent the successfully matched descriptors from the current frame and the loop frame, respectively.

For the FusionPortable dataset, we evaluate four representative sequences, with a focus on two challenging cases: building_day and vehicle_campus01. In the building_day complex indoor environment, the keypoint selection strategy of STD becomes unstable, leading to numerous mismatches or matching failures (as is shown in Figure 6). In contrast, DTD benefits from density-based keypoint extraction, leading to a two-fold improvement in F1 max score over STD. In vehicle_campus01, similar road structures and limited height variation make Scan Context prone to false detections; specifically, Figure 5l visually demonstrates that Scan Context maintains relatively low loop closure performance, and Table 2 shows that STD improves the F1 max score by 68.04% compared to Scan Context. DTD maintains high performance owing to its robust keypoint extraction and entropy-based similarity evaluation.

### 4.3. Ablation Study

In Section 3.2, we introduced the entropy-based similarity function. To evaluate the contribution of entropy to the local descriptor, we conduct an ablation study to analyze its impact during the loop detection phase. Specifically, DTD (w/o entropy) refers to the variant that removes the entropy validation and performs matching solely based on triangle side lengths. As shown in Figure 5 and Table 2, incorporating entropy significantly improves the recall rate of DTD and achieves better F1 max score and EP. This improvement is consistent across all sequences, demonstrating strong robustness and stability.

**Figure 7:**
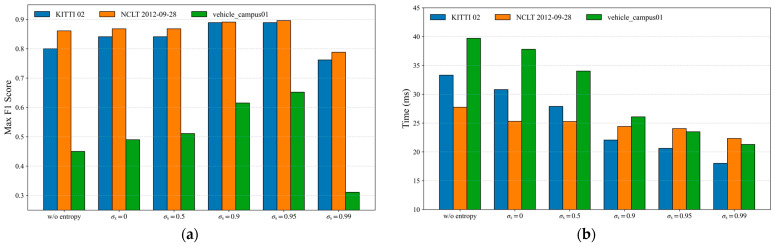
F1 max score (**a**) and average computation time (**b**) of DTD on the KITTI 02, NCLT 2012-09-28, and vehicle_campus01 sequences under different similarity thresholds  $\sigma_{s}$.

Furthermore, the proposed entropy-based local descriptor effectively filters out incorrect loop candidates, accelerating the overall matching process while improving loop detection performance. To evaluate its contribution, we select representative sequences from the KITTI, NCLT, and FusionPortable datasets (KITTI 02, NCLT 2012-09-28, and vehicle_campus01) and conduct comparative experiments. Two metrics are adopted: the F1 max score and the total processing time. These metrics are used to (1) verify the performance gains brought by the introduction of entropy and (2) analyze the influence of different similarity thresholds  $\sigma_{s}$ after incorporating entropy-based verification, as shown in Figure 7.

Figure 7 shows the variations in F1 max scores and computation time under DTD (w/o entropy) and DTD with various similarity thresholds  $\sigma_{s}$. Experimental results indicate that, after incorporating entropy verification, DTD achieves higher F1 max scores and lower time consumption than DTD (w/o entropy) even when  $\sigma_{s}$ = 0. Specifically, when  $\sigma_{s}$ = 0.95, the F1 max scores of these three sequences increased by 11.13%, 4.07%, and 44.89%, respectively, while the computation time decreased by 38.13%, 13.19%, and 40.84%. This demonstrates that although entropy computation introduces an additional cost, it can rapidly eliminate false candidates with similar density-triangle structures, thereby reducing the overall matching time.

Moreover, the choice of  $\sigma_{s}$ affects the performance of DTD. Generally, a larger threshold leads to better detection results; however, when  $\sigma_{s}$ approaches 1, correct loop candidates may be mistakenly discarded. Therefore, in practical applications, $\sigma_{s}$ is typically set within the range of 0.90–0.95 to balance accuracy and robustness.

### 4.4. Performance on LiDAR-Based SLAM

**Figure 8:**
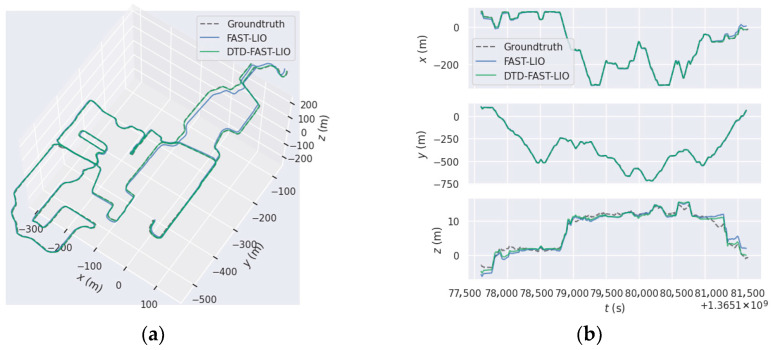

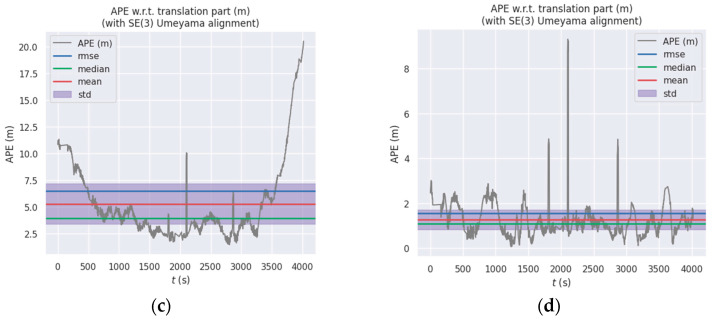
Example on NCLT 2013-04-05: (**a**,**b**) comparison of estimated and ground-truth trajectories for FAST-LIO and DTD-FAST-LIO; (**c**,**d**) Absolute Pose Error (APE) profiles of FAST-LIO (**c**) and DTD-FAST-LIO (**d**).

In this experiment, we evaluate the effectiveness of the proposed loop closure detection module within a LiDAR SLAM system. DTD, STD, and Scan Context are, respectively, integrated into FAST-LIO (denoted as DTD-FAST-LIO, STD-FAST-LIO, and SC-FAST-LIO) and tested on the NCLT and FusionPortable datasets. To quantify the impact of loop closure on trajectory accuracy, we use the EVO toolkit to compute the Absolute Pose Error (APE) and adopt Root Mean Square Error (RMSE) as the evaluation metric, where a lower RMSE indicates better global consistency. The trajectory optimization results are presented in Figure 8 and Table 3.

As shown in Table 3, compared with the original FAST-LIO system, DTD-FAST-LIO consistently yields lower RMSE values across almost all tested sequences, indicating that the proposed loop closure module effectively suppresses accumulated drift. Taking the NCLT dataset as an example, the average RMSE decreased by 51.02% after incorporating DTD. Further comparison with STD-FAST-LIO and SC-FAST-LIO demonstrates that DTD achieves the lowest RMSE on all sequences, validating the superiority of the proposed method.

Moreover, the trajectory comparison in Figure 8 visually illustrates that DTD successfully identifies loop closures and significantly improves accumulated drift. Overall, the proposed loop closure detection module can be integrated into existing LiDAR SLAM frameworks and substantially improves global pose estimation accuracy.

### 4.5. Computational Complexity

**Figure 9:**
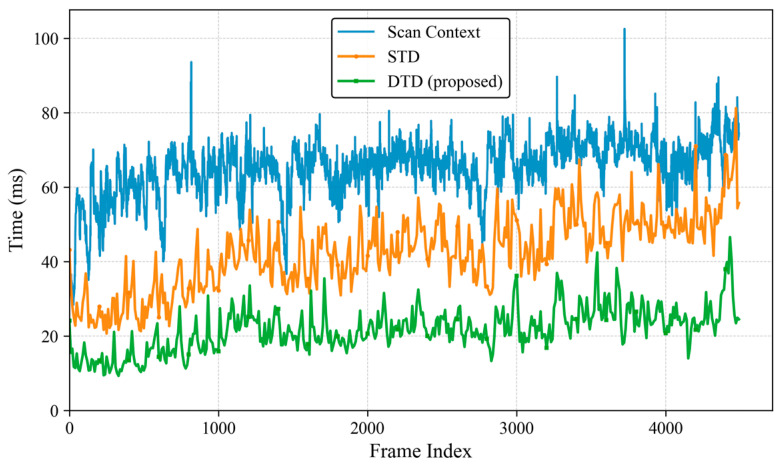
Runtime Evaluation on KITTI 00.

In this section, we evaluate the average computational time of different loop detection methods, the results are presented in Figure 9. Table 4 presents the runtime comparison of each submodule on the KITTI 00 sequence. For Scan Context, we used the official MATLAB (R2022b) implementation with default parameters, while both STD and DTD were implemented in C++.

The results show that Scan Context exhibits the highest computational cost. In contrast, the total processing time of DTD is reduced by 50.57% and 73.85% compared to STD and Scan Context, respectively, which is mainly due to the keypoint selection strategy based on the density map. Although the introduction of local descriptors slightly increases the time required to insert descriptors into the database, this overhead is negligible considering the improved robustness achieved. As illustrated in Figure 9, the overall runtime of DTD remains stable as the number of frames increases, demonstrating its clear advantage in terms of real-time performance.

### 4.6. Applicability to Different LiDAR Types

This section mainly evaluates the applicability of the proposed DTD method to different types of LiDAR sensors. We use two solid-state LiDARs, Avia and Aeva, provided by the HeLiPR dataset. These sensors have different Fields of View (FOVs) and employ non-repetitive scanning patterns, as detailed in Table 1.

**Figure 10:**
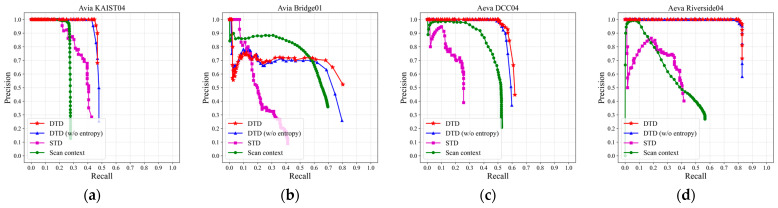
Precision–recall curves for HeLiPR dataset: (**a**,**b**) captured by Livox Avia LiDAR; (**c**,**d**) captured by Aeva Aeries II LiDAR.

In the experimental setup, we accumulate 10 frames as a keyframe, and a detected loop is regarded as a true positive when the GT distance is less than 15 m. The remaining algorithmic parameters are consistent with those described in Section 4.2. The corresponding PR curves and loop closure performance metrics are shown in Figure 10 and Table 5, respectively.

As can be observed from Table 5, DTD achieves higher F1 max scores and EP values than STD and Scan Context on almost all sequences. Compared with STD, the F1 max scores are improved by 24%, 116.61%, 95.03%, and 86.61%, respectively, indicating that DTD maintains strong loop closure performance under solid-state LiDAR data. In addition, DTD consistently outperforms DTD (w/o entropy) across all sequences, with F1 max scores increasing by 3.33%, 5.02%, 3.07%, and 0.79%, further verifying the effectiveness of the entropy-based local descriptor in enhancing loop closure detection performance.

**Figure 11:**
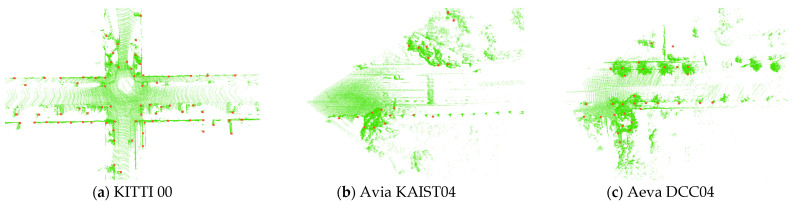
Comparison of DTD keypoint extraction results under different LiDAR types, the red dots denote the keypoints.

To intuitively analyze the impact of different LiDAR types on DTD, we compare the keypoint extraction results on the KITTI 00, Avia KAIST04, and Aeva DCC04 datasets, as shown in Figure 11. Since KITTI 00 is collected using a LiDAR sensor with a 360° FOV, it has the richest set of keypoints, while Avia and Aeva have smaller FOVs but higher point cloud densities. The experimental results show that the density-image-based keypoint extraction strategy remains stable under different sensor conditions and effectively reflects regions with higher point cloud density.

In summary, DTD is not only applicable to traditional mechanically rotating LiDARs but can also be applied to non-repetitive scanning LiDARs, demonstrating good generality and adaptability.

## 5. Discussion

As shown in Figure 5 and Table 2, DTD demonstrates superior overall performance in loop closure detection compared with STD and Scan Context, achieving higher F1 max score and EP across most sequences. Further analysis reveals that DTD exhibits strong robustness across diverse scenarios: on multiple sequences from the NCLT and FusionPortable datasets, the comparison methods suffer from varying degrees of performance degradation. Specifically, Scan Context only uses the maximum height of the point cloud for projection, which easily leads to mismatches in open or scenes with insignificant height variations; STD directly extracts corner points from the 3D point cloud as keypoints, resulting in keypoints being more concentrated near the lidar, as intuitively shown in Figure 6. In contrast, inspired by previous BEV image-based feature extraction methods (such as BEVPlace [32]), DTD extracts keypoints based on density images, effectively covering longer distances and areas with richer structures, demonstrating stronger adaptability to complex environments.

In addition, Figure 5 and Table 2 further verify the effectiveness of the proposed entropy-based local descriptor. Similar to probabilistic or information-theoretic modeling methods used in previous loop detection research (such as NDD [24]), entropy can effectively characterize the distribution characteristics of point clouds. By incorporating entropy into DTD, more reliable similarity evaluation for candidate loop closures can be achieved. The ablation study results presented in Figure 7 demonstrate that this local verification strategy not only improves the F1 and EP metrics, but also significantly reduces the computational cost of loop detection by filtering out incorrect candidate frames early.

Furthermore, DTD is integrated into a real-world LiDAR odometry system, FAST-LIO. As shown in Figure 8 and Table 3, the introduction of loop closure detection effectively reduces accumulated drift and improves global trajectory consistency. Although STD and Scan Context can also improve trajectory quality to some extent, the results in Table 3 show that DTD still has a more significant advantage in the RMSE metric, which is consistent with the conclusions drawn from the loop closure detection experiments.

Figure 9 and Table 4 highlight the real-time performance advantages of DTD. Statistical analysis of the runtime of the submodules shows that compared to STD, the efficiency improvement of DTD mainly comes from its descriptor extraction strategy and two-stage loop detection method. Keypoint extraction based on density images is more efficient than directly operating on 3D point clouds, while the entropy-based local descriptor reduces unnecessary matching computations, thereby improving overall system efficiency.

Finally, we evaluate the applicability of DTD under other types of LiDAR. The results in Figure 10 and Table 5 show that DTD consistently outperforms the comparison methods under different LiDAR types and scanning modes. This is mainly due to the keypoint extraction strategy employed: as shown in Figure 11, even when the sensor type and acquisition method change, the keypoints extracted by DTD can still reliably reflect the high-density structural regions in the point cloud.

## 6. Conclusions

This paper proposes a novel global descriptor, the Density Triangle Descriptor (DTD), for LiDAR loop closure detection in complex environments. DTD consists of keypoint extraction based on density images, descriptor construction, and a two-stage loop closure detection strategy. By extracting keypoints from density BEV images, this method outperforms direct manipulation of 3D point clouds in terms of efficiency and stability. Simultaneously, an entropy-based local descriptor is introduced to enhance robustness to local structural changes, and geometric constraints are combined to achieve reliable loop closure verification and pose estimation.

The method is systematically validated on four public datasets: KITTI, NCLT, FusionPortable, and HeLiPR, covering various complex scenarios and different types of LiDAR sensors. Compared to STD and Scan Context methods, DTD demonstrates significant advantages in both loop closure detection accuracy and computational efficiency. Experimental results show that compared to STD, DTD improves the average F1 max score and EP by 18.30% and 20.08%, respectively, while increasing computational efficiency by 50.57%. Furthermore, integrating DTD into the FAST-LIO odometry system effectively suppresses long-term accumulated errors, reducing the trajectory RMSE by an average of 51.02%. These results demonstrate that DTD possesses good stability, robustness, and environmental adaptability in complex environments, validating its potential for application in practical SLAM systems.

In future work, we will further expand the descriptor’s expressive power and application scope. We will consider introducing richer keypoint description information (such as semantic features) to improve the ability to characterize complex scene structures. In addition, we will explore multi-sensor fusion strategies, introducing visual information in LiDAR degradation scenarios to provide additional constraints to the system, further improving the system’s robustness and applicability.

## Figures and Tables

**Table 1:** The details of the datasets used.

Dataset	KITTI	NCLT	FusionPortable	HeLiPR	
Sensor	Spinning	Spinning	Spinning	Solid state	
Scanning Pattern	Repetitive	Repetitive	Repetitive	Non-Repetitive	
LiDAR	Velodyne HDL-64E	Velodyne HDL-32E	Ouster OS1-128	Livox Avia	Aeva Aeries II
Field of View	360° × 26.8°	360° × 41.3°	360° × 45°	70° × 77°	120° × 19.2°
Sequence	00	2012-05-26	ugv_transition00	KAIST04 Bridge01	DCC04 Riverside04
	02	2012-08-20	ugv_parking01		
	05	2012-09-28	building_day		
	08	2013-04-05	vehicle_campus01		
Environment	Urban	Campus	Unstructured, Indoor	Campus, Bridge	

**Table 2:** The F1 max scores and extended precision on datasets.

Sequence	DTD		DTD (w/o Entropy)		STD		Scan Context	
	F1 Max	EP	F1 Max	EP	F1 Max	EP	F1 Max	EP
KITTI 00	0.962	0.947	0.855	0.874	**0.985**	**0.985**	0.962	0.963
KITTI 02	**0.889**	**0.897**	0.800	0.833	0.815	0.813	0.810	0.450
KITTI 05	**0.976**	**0.976**	0.958	0.960	0.964	0.964	0.948	0.939
KITTI 08	**0.989**	**0.989**	0.978	0.978	0.939	0.814	0.761	0.563
NCLT 2012-05-26	**0.879**	**0.850**	0.850	**0.850**	0.840	0.612	0.766	0.503
NCLT 2012-08-20	**0.836**	**0.845**	0.816	0.839	0.729	0.595	0.733	0.501
NCLT 2012-09-28	**0.896**	**0.884**	0.861	0.863	0.618	0.562	0.771	0.517
NCLT 2013-04-05	**0.912**	**0.919**	0.836	0.855	0.628	0.532	0.709	0.536
ugv_transition00	**0.923**	**0.929**	0.909	0.916	0.884	0.643	0.744	0.752
ugv_parking01	**0.908**	**0.833**	0.879	0.800	0.569	0.567	0.649	0.689
building_day	**0.674**	**0.681**	0.633	0.638	0.222	0.521	0.664	0.611
vehicle_campus01	**0.652**	0.643	0.450	0.589	0.526	**0.679**	0.388	0

Note: The best results are marked in bold and the second-best results are underlined.

**Table 3:** The APE (meters) of FAST-LIO with different loop closure configurations on various datasets.

Sequence	FAST-LIO		DTD-FAST-LIO		STD-FAST-LIO		SC-FAST-LIO	
	RMSE	Mean	RMSE	Mean	RMSE	Mean	RMSE	Mean
NCLT 2012-05-26	2.382	2.066	**1.574**	1.346	1.608	1.386	1.635	1.402
NCLT 2012-08-20	2.288	2.064	**1.598**	1.399	1.675	1.450	1.618	1.387
NCLT 2012-09-28	2.930	2.231	**1.057**	0.923	1.188	1.030	1.931	1.408
NCLT 2013-04-05	6.474	5.272	**1.550**	1.273	2.978	2.529	3.322	2.816
ugv_transition00	**0.201**	0.147	0.201	0.147	0.203	0.149	0.201	0.147
ugv_parking01	0.382	0.332	**0.359**	0.312	0.386	0.331	0.363	0.318
building_day	1.560	1.333	**1.514**	1.241	1.531	1.258	1.545	1.308
vehicle_campus01	5.049	3.971	**5.047**	3.968	5.050	3.971	5.053	3.973

Note: The best results are marked in bold.

**Table 4:** Average computational time of different submodules on KITTI 00.

Method	Time Consumption (ms)			
	Descriptor Construction	Loop Detection	Add to Database	Total
Scan Context	47.063	30.289	**0.017**	77.517
STD	27.249	13.661	0.099	41.009
DTD	**13.498**	**6.573**	0.198	**20.269**

Note: The best results are marked in bold.

**Table 5:** The F1 max scores and extended precision on HeLiPR dataset.

Sequence	DTD		DTD (w/o Entropy)		STD		Scan Context	
	F1 Max	EP	F1 Max	EP	F1 Max	EP	F1 Max	EP
Avia KAIST04	**0.620**	**0.724**	0.600	0.714	0.500	0.609	0.419	0.593
Avia Bridge01	**0.691**	0.508	0.658	0.506	0.319	**0.536**	0.637	0.500
Aeva DCC04	**0.706**	**0.751**	0.685	0.743	0.362	0.508	0.570	0.501
Aeva Riverside04	**0.892**	**0.901**	0.885	0.891	0.478	0.506	0.452	0.500

Note: The best results are marked in bold and the second-best results are underlined.

## Data Availability

Publicly available datasets were analyzed in this study. These data can be found here: KITTI dataset: https://www.cvlibs.net/datasets/kitti/eval_odometry.php (accessed on 1 November 2025); NCLT dataset: http://robots.engin.umich.edu/nclt/ (accessed on 1 November 2025). FusionPortable Dataset: https://fusionportable.github.io/ (accessed on 1 November 2025). HeLiPR Dataset: https://sites.google.com/view/heliprdataset/download (accessed on 1 November 2025). EVO tool: https://github.com/MichaelGrupp/evo (accessed on 1 November 2025).
